# The impact of inactivity during the COVID-19 pandemic on the physical performance of high school athletes

**DOI:** 10.1186/s13102-024-00916-3

**Published:** 2024-06-06

**Authors:** Selim Asan, Süleyman Ulupınar, Serhat Özbay, Sevinç Namlı, Cebrail Gençoğlu, Ferhat Canyurt, Yunus Emre Çingöz, Abdullah Bora Özkara

**Affiliations:** 1 Faculty of Sport Sciences, Erzurum Technical University, Erzurum, Turkey; 2 Faculty of Sport Sciences, Bayburt University, Bayburt, Turkey; 3 Faculty of Sport Sciences, Karadeniz Technical University, Trabzon, Turkey

**Keywords:** COVID-19 pandemic, Physical activity, School athletes, Health-Related Fitness, ALPHA Test

## Abstract

**Background/Objective:**

This study was conducted during the 2019–2020 academic year to evaluate the impact of participation in school sports on students’ Body Mass Index (BMI) and Assessing Levels of Physical Activity (ALPHA) test scores. Interrupted by the COVID-19 pandemic, which led to a suspension of in-person education, the study resumed in September 2021, refocusing on the effects of pandemic-induced inactivity on the physical fitness levels of the same cohort.

**Methods:**

The study included twenty-nine male high school students (age: 17,24 ± 0,73 years), divided into thirteen athletes (participating in sports such as football, basketball, and track) and sixteen non-athletic counterparts. They underwent reassessment using the ALPHA test battery, evaluating cardiorespiratory, musculoskeletal, and motor skills fitness. Data were analyzed using independent and paired samples t-tests and a two-way repeated measures ANOVA to assess changes over time and between groups. Discriminant function analysis evaluated the ALPHA test’s ability to classify students based on their athletic status pre- and post-pandemic.

**Results:**

Initially, athlete students exhibited significantly better BMI, 20 m shuttle run, and 4 × 10 m speed run scores compared to their non-athlete peers. After the pandemic, only the 20 m shuttle run scores remained significantly higher for athletes, with diminished distinctions in other fitness areas. The classification accuracy of the ALPHA test battery decreased from 89.7 to 75.9% post-pandemic.

**Conclusion:**

The enforced sedentary lifestyle due to the COVID-19 pandemic adversely affected all students, particularly diminishing health-related fitness parameters such as body composition, cardiorespiratory and musculoskeletal strength, and motor skills. Students previously engaged in regular physical activity, notably school athletes, experienced significant fitness declines. This highlights the urgent need for targeted interventions to encourage active lifestyles among youth in the post-pandemic phase, aiming to avert long-term adverse health outcomes.

## Background

In the contemporary era, the limitations imposed by time and space have increasingly influenced the lifestyles and activity levels of children and adolescents [1, 2]. Schools have traditionally played a pivotal role in fostering physical activity among young people, acting not only as educational institutions but also as environments conducive to health and physical well-being [3, 4]. However, the emergence of the COVID-19 pandemic has significantly disrupted conventional schooling methods, leading to a marked rise in sedentary behavior among the youth due to the widespread suspension of face-to-face educational activities [5, 6]. This interruption of routine school activities, especially physical education and sports, has resulted in a loss of critical opportunities for engaging in physical activity, thereby fostering a sedentary lifestyle that detrimentally impacts both physical and mental health [4, 7, 8].

Conversely, for those who had already adopted a sedentary lifestyle prior to the pandemic’s outbreak, the changes brought about by COVID-19 may not seem markedly significant [8–10]. This group, already habituated to a lifestyle with minimal physical activity, might not view the pandemic-induced restrictions and home confinement as a drastic deviation from their typical routines. On the contrary, individuals who led active lifestyles before the pandemic, particularly students involved in school sports and physical activities, have experienced significant disruptions [8, 11]. Accustomed to regular exercise, competitions, and the sociability of team sports, these students have faced abrupt cessation of their usual physical and social activities. The transition from active engagement to enforced sedentary living due to lockdown measures has posed a substantial challenge, likely resulting in pronounced declines in their physical fitness, mental well-being, and overall health [8, 12].

The differential impact of the pandemic on physical activity levels thus highlights a divergence: while individuals with previously sedentary lifestyles may have observed little change, those who were actively engaged, especially in school sports, have encountered significant obstacles [8, 11]. This divergence underlines the critical need for specialized strategies to counteract the varied effects of the pandemic across different population segments. For students formerly active in sports, reengagement in physical activities and reestablishment of their former participation levels are essential for their comprehensive post-pandemic recovery. Consequently, assessing how the pandemic has differently impacted students involved in school sports and their non-athletic counterparts is vital. This evaluation serves as a crucial foundation for the development of specific interventions designed to alleviate the adverse effects associated with extended periods of inactivity. Therefore, the primary goal of this research is to investigate the disparate impacts of the COVID-19 pandemic on the physical activity levels and health-related fitness parameters between high school students who were actively involved in school sports prior to the pandemic and those who maintained a sedentary lifestyle.

## Methods

### Research design

The present study employed a mixed-method approach to examine the differential impacts of the COVID-19 pandemic on high school students, distinguishing between those actively participating in sports and their sedentary peers. Utilizing a longitudinal framework, we conducted assessments before and after the pandemic’s onset, facilitating a detailed comparison of temporal changes in physical fitness levels. Our primary tool for evaluation was the ALPHA test battery, which allowed us to quantitatively measure vital health-related physical fitness parameters, including cardiorespiratory fitness, musculoskeletal strength, and motor skills [13, 14]. This design enabled a nuanced analysis of how the pandemic has affected the physical health of these two distinct student groups.

### Participants

This research was conducted with a group of 29 male high school students, each aged between 16 and 18 years old. These students had progressed to secondary education prior to the COVID-19 pandemic and remained enrolled through September 2021. Initial measurements were taken between October and November 2019, with the final assessments completed in September 2021. Originally, the purpose of these initial measurements was distinct and not primarily focused on the pandemic’s impact. However, the onset of COVID-19 significantly shifted our research focus, allowing us to utilize these pre-existing data points to investigate the pandemic’s effects on the physical fitness of these students over the specified period. Initially, the research commenced with 68 students prior to the pandemic, but post-pandemic conditions resulted in only 29 students completing the study. Participants were divided into two groups: 13 students actively involved in at least one school sports team (four in football, three in basketball, four in volleyball, two in table tennis) and 16 students not engaged in any sports activities. The selection of participants was based on voluntariness, ensuring both groups were homogeneous in terms of age, gender, and educational level.

Before the pandemic, these participants engaged in training sessions 3 to 5 times per week, with each session lasting between 90 and 120 min. The majority of these sessions consisted of aerobic exercises, but they also included sport-specific training such as plyometrics, sprints, agility drills, and calisthenic strength development exercises. Prior to participating in the study, all participants and their parents signed informed consent forms, which detailed the purpose, procedures, potential benefits, and risks of the study. Participants were also required to attend a structured trial session before the tests, during which they were fully informed about the study’s procedures and tests. Inclusion criteria were defined as being enrolled in secondary education during the study period and being actively involved in school sports teams before the pandemic or not participating in any sports activities. Exclusion criteria included students who did not regularly attend school during the study period, were unable to participate in the tests due to health problems, or did not sign the informed consent form.

### Data collection

Height and body mass measurements were conducted with precision using standardized equipment. Heights were measured to the nearest 0.1 cm using a stadiometer (Holtain Ltd., Crosswell, Crymych, Dyfed, United Kingdom). Body weights were assessed to the nearest 0.1 kg on a validated digital scale, ensuring reliable data for Body Mass Index (BMI) calculations and other health-related assessments.

The ALPHA test battery was utilized to evaluate a range of physical fitness metrics. The 20-meter shuttle run test was conducted to estimate maximal oxygen uptake (VO_2max_), an essential measure of cardiorespiratory endurance. This test involves participants running between two lines 20 m apart, keeping pace with audio beeps that increase in speed at set intervals. The VO_2max_ estimation utilized the formula: VO_2max_ = 0.301 × (shuttle run score) − 0.9 × (age) − 6.642 × (gender) − 0.173 × (body weight in kg) + 63.168, where gender is assigned values (1 for males, 0 for females), providing a significant assessment of cardiorespiratory health [13]. Musculoskeletal fitness was assessed using the standing long jump test for lower body strength and the handgrip strength test for upper body strength (Takei A5001 Hand Grip Dynamometer). The participants’ agility and motor coordination were assessed through the 4 × 10-meter shuttle run, requiring swift direction changes and speed. Performance times were accurately recorded using a photoelectric cell system, ensuring exact measurement of each participant’s agility (Smart Speed electronic system, Fusion Sport, Cooper Plains, Australia).

### Statistical analyses

Data analysis was performed using IBM SPSS Statistics for Windows, Version 25.0 (IBM Corp, Chicago, IL, USA). All data were reported as mean ± standard deviation, with a significance level set at *p* ≤ 0.05. Initial differences between groups were analyzed using independent samples t-tests, while within-group changes were evaluated using paired samples t-tests, with effect sizes presented. Effect sizes were calculated using Cohen’s d formula [15, 16] and classified according to Hopkins [16, 17], aiding in the assessment of the practical significance of the study’s findings. To examine the interactions between time and group, a two-way (group×time) repeated measures ANOVA was utilized. This statistical approach allowed for the examination of changes over time within each group and the differences between groups over the same period. To address the challenges posed by our study’s small sample size and enhance the reliability of our findings, we employed bootstrap methods in conducting the discriminant function analysis. Specifically, we utilized the bootstrap technique with 1000 samples, coupled with Bias Corrected and Accelerated (BCa) confidence intervals, to robustly estimate the classification accuracy of the model. This approach allowed us to more accurately predict group membership based on the independent variables of the ALPHA test battery, while also mitigating potential biases and improving the precision of our estimates.

## Results

In the athlete group, excluding handgrip strength, all parameters showed a significant decrease in performance post-pandemic compared to pre-pandemic levels (Table 1). BMI, 20 m shuttle run, and Standing Broad Jump variables exhibited a moderate effect size, indicating a significant reduction in these fitness measures due to the pandemic. The 4 × 10-meter shuttle run variable demonstrated a large effect size, highlighting a more pronounced decrease in agility performance within the athlete group after the pandemic. The sedentary group displayed a significant decrease only in the 20 m shuttle run post-pandemic, with a moderate effect size.

**Table 1:** Comparative Analysis of Pre- and Post-Pandemic Physical Fitness Measures in Athlete and Sedentary High School Male Students

		Pre-test	Post-test	d
Athlete group	Body Mass Index (kg.m^− 2^)	21.7 ± 1.3^**†**^	23.2 ± 2.0*	0.89
	20-meter Shuttle Run (VO_2max_ ml.kg^− 1^.min^− 1^)	50.5 ± 2.1^**†**^	48.1 ± 2.2*^❖^	1.12
	Standing Broad Jump (cm)	163.7 ± 8.1	157.9 ± 6.5*^#^	0.79
	Handgrip Strength (kg)	32.7 ± 3.3	32.2 ± 2.7	0.17
	4 × 10-meter Shuttle Run (sec)	11.2 ± 0.3^**†**^	11.9 ± 0.6*	1.48
Sedentary group	Body Mass Index (kg.m^− 2^)	23.4 ± 2.4	23.5 ± 1.8	0.05
	20-meter Shuttle Run (VO_2max_ ml.kg^− 1^.min^− 1^)	46.8 ± 2.3	45.6 ± 1.6*	0.61
	Standing Broad Jump (cm)	160.4 ± 9.3	159.8 ± 7.0	0.07
	Handgrip Strength (kg)	32.1 ± 2.8	31.8 ± 2.5	0.11
	4 × 10-meter Shuttle Run (sec)	11.9 ± 0.6	11.9 ± 0.6	0.00

Values are presented as mean ± standard deviation. d = effect size. * indicates a difference in the post-pandemic period compared to the pre-pandemic period. ^**†**^ indicates that the athlete group performed better in the pre-test compared to the sedentary group. ^**❖**^ indicates that the athlete group performed better in the post-test compared to the sedentary group. ^**#**^ indicates a significant group*time interaction, indicating that the change over time in the athlete group was greater than in the sedentary group

Pre-pandemic, the athlete group significantly outperformed the sedentary group in Body Mass Index, 20 m shuttle run, and 4 × 10-meter shuttle run parameters, indicating higher fitness levels among the athletes. However, post-pandemic, only the 20 m shuttle run parameter remained significantly higher in the athlete group compared to the sedentary group, suggesting a retained but diminished advantage in cardiorespiratory fitness. Furthermore, a significant group*time interaction was observed in the Standing Broad Jump parameter. This finding indicates that the decline in performance due to the pandemic’s impact was greater in the athlete group than in the sedentary group, highlighting the more substantial effect of reduced physical activity and altered training conditions on students previously engaged in regular athletic activities.

Discriminant function analysis was employed to classify the participants into either the athlete or sedentary group based on their pre-pandemic physical fitness measures (Table 2). The analysis accurately classified 89.7% of the students into their correct groups, demonstrating the efficacy of the physical fitness measures in distinguishing between the two lifestyles. Specifically, for the athlete group, consisting of 13 members, 92.3% (12 students) were correctly classified into the athlete group, while 7.7% (1 student) was mistakenly classified into the sedentary group. On the other hand, in the sedentary group, which comprised 16 members, 81.2% (13 students) were correctly identified as part of the sedentary group, and 18.8% (3 students) were incorrectly classified as belonging to the athlete group.

**Table 2:** Classification of Groups According to Pre-Pandemic Physical Fitness Measurements Using Discriminant Function Analysis

Original groups	*n*	Estimated assigned group memberships	
		Athlete group	Sedentary group
Athlete group	13	92.3% (12)	7.7% (1)
Sedentary group	16	12.5% (2)	87.5% (14)

Prior to the pandemic, the classification accuracy of the ALPHA test battery was 89.7%

**Table 3:** Classification of Groups According to Post-Pandemic Physical Fitness Measurements Using Discriminant Function Analysis

Original groups	*n*	Estimated assigned group memberships	
		Athlete group	Sedentary group
Athlete group	13	69.2% (9)	30.8% (4)
Sedentary group	16	18.8% (3)	81.2% (13)

After the pandemic, the classification accuracy of the ALPHA test battery was 75.9%

Utilizing discriminant function analysis to classify participants based on their post-pandemic physical fitness measures yielded an overall correct classification rate of 75.9% (Table 3). Among the athlete group, which consisted of 13 individuals, 69.2% (9 students) were accurately classified back into the athlete group, whereas 30.8% (4 students) were mistakenly classified as part of the sedentary group. This misclassification indicates a notable impact of the pandemic on the physical fitness levels of students previously identified as athletes, affecting their classification based on post-pandemic physical fitness measures. In the sedentary group, comprising 16 individuals, 81.2% (13 students) were correctly identified as sedentary, while 18.8% (3 students) were inaccurately classified as belonging to the athlete group. This classification accuracy demonstrates a relative stability in the physical fitness measures of the sedentary group when compared to the pre-pandemic period.

The discriminant function analysis conducted for pre-pandemic and post-pandemic physical fitness measures utilized Box’s M statistic to assess the equality of covariance matrices across groups. For the pre-pandemic analysis, Box’s M value was 20.305, with an associated F value of approximately 1.074 (df_1_ = 15, df_2_ = 2649.3), resulting in a significance level of 0.375. In the post-pandemic analysis, Box’s M value increased to 26.474, with an F value of approximately 1.401 (df_1_ = 15, df_2_ = 2649.3), and a significance level of 0.138. The significance levels in both analyses (0.375 pre-pandemic and 0.138 post-pandemic) suggest that the assumption of equal covariance matrices across groups was not violated, indicating that the discriminant function analysis was appropriately applied to the data. Furthermore, the analysis confirmed the absence of multicollinearity among the ALPHA test variables, with the highest correlation coefficient (r) observed being 0.62. This indicates that the variables within the ALPHA test battery were sufficiently independent, ensuring the validity of the discriminant function analysis results.

## Discussion

The COVID-19 pandemic has significantly disrupted the daily lives and routines of individuals worldwide, with particularly profound effects on the physical activity and fitness levels of adolescents [2, 6, 18]. This study provides an in-depth analysis of these impacts, highlighting the varied experiences of high school students engaged in athletic programs compared to their sedentary peers. Our findings indicate a broad decline in physical fitness across most parameters for the athlete group, an observation that starkly contrasts with the relatively stable metrics, apart from the 20 m shuttle run, in the sedentary group. This disparity underscores the extensive disruption experienced by students typically involved in structured physical activities, emphasizing the far-reaching consequences of the pandemic on established fitness routines.

In light of global trends, comprehensive research utilizing Argus app data demonstrates a significant worldwide reduction in physical activity, as reflected by decreased daily step counts following pandemic-induced restrictions [18]. This global phenomenon provides a background for our findings, reinforcing the dramatic declines in specific fitness parameters among high school athletes we observed. The universal challenge posed by social distancing measures and lockdowns is further exemplified by the nuanced perspectives our study offers. The differential impacts we observed—substantial declines in endurance and agility for athletes versus minimal changes for the sedentary—echo the broader disruptions documented globally. Additionally, another study details an international online survey initiated seven languages to illuminate the behavioral and lifestyle consequences of COVID-19 restrictions [10]. This report presents critical results on physical activity and nutrition behaviors, indicating a negative effect on all intensities of physical activity with an increase in daily sitting time from 5 to 8 h per day. These findings suggest that while isolation is a necessary measure to protect public health, it alters physical activity and eating behaviors in a direction that could compromise health. This broader context supports our specific observations of dramatic declines in fitness parameters among high school athletes, providing a deeper understanding of the pandemic’s impact on young individuals engaged in regular sports activities.

The findings of our study align with the longitudinal research conducted in China in several aspects, indicating broad impacts of the COVID-19 pandemic on adolescent physical fitness [8]. However, a notable divergence is observed in the realm of muscular strength. While Zhou et al. (2022) reported an improvement in this area among adolescents during the lockdown, reflected through enhanced performance in exercises like pull-ups, our study did not observe similar trends. Specifically, handgrip strength among our participants remained unchanged, underscoring a distinct contrast in the outcomes related to muscular strength. This difference may underscore the variation in the effectiveness of different types of home-based physical activities undertaken during lockdown conditions [19, 20]. It suggests that pandemic impacts on physical fitness components vary, with the nature and type of activities available during lockdowns significantly influencing specific fitness outcomes. This observation emphasizes the complex nature of physical fitness adaptations under pandemic-induced restrictions, highlighting the need for diverse and adaptable physical activity strategies [21, 22].

The notable decrease in physical fitness among adolescents with higher baseline fitness levels, a trend consistent with both our findings and the previously mentioned studies [8, 10, 12], underscores the uneven impact of the pandemic across different demographic groups. The pandemic has not only disrupted regular physical activity but also led to significant lifestyle shifts, including dietary changes, as individuals, including athletes, navigate the constraints of lockdowns and social distancing [1, 9, 23, 24]. The increase in sedentary behaviors and the shift towards unhealthy dietary patterns, exacerbated by the pandemic as highlighted by previous research, have contributed to the declining physical fitness levels observed [24, 25]. This shift is concerning and suggests a compounded effect of reduced physical activity and poor nutrition on overall adolescent health and well-being.

The necessity of regular exercise, emphasized by existing studies, becomes particularly pertinent in light of our findings. Despite pandemic-related challenges, the significance of engaging in home-based exercises such as yoga, pilates, and aerobics cannot be overstated in countering the effects of prolonged inactivity [21, 26, 27]. The observed post-pandemic decrease in physical activity rates, coupled with our research outcomes, underscores the urgent need for specialized interventions that foster physical activity and nutritional well-being during periods marked by significant restrictions [19, 23, 25]. In conclusion, our study contributes valuable insights into the specific effects of the COVID-19 pandemic on the physical fitness of adolescents, highlighting the critical need for strategies to support youth health during times of global crisis. By documenting the decline in physical fitness among athletes and noting the relative stability among sedentary peers, we underscore the pandemic’s broad impact and the importance of maintaining an active lifestyle and healthy diet, even under challenging conditions. Moving forward, it is imperative to develop and implement comprehensive public health strategies that can effectively address the multifaceted challenges presented by the pandemic, ensuring the physical and mental well-being of adolescents in these unprecedented times.

### Limitations of the study and recommendations for Future Research

While our study offers significant insights into the impacts of the COVID-19 pandemic on adolescent physical fitness, several limitations must be acknowledged to better interpret and generalize the findings. First, the sample size and demographic scope of our study, primarily focusing on male high school students from a single geographical region, may limit the generalizability of our findings to broader populations. Additionally, as the study involved only one type of educational setting, future studies should aim to include a more diverse cohort encompassing different age groups, genders, and socio-economic backgrounds. This expansion would provide a more comprehensive understanding of the pandemic’s impact on physical fitness across various demographics. Moreover, our study assessed conventional fitness metrics such as cardiorespiratory fitness, musculoskeletal strength, and motor skills, without delving into psychological or behavioral changes that might accompany altered physical fitness levels. Acknowledging the interrelation between physical and psychological health, future research should consider these aspects to provide a holistic view of adolescents’ well-being during and after the pandemic. The longitudinal design of our study was also constrained by the unique circumstances of the pandemic, which limited our ability to conduct follow-up assessments under normal conditions. As such, long-term studies extending beyond the pandemic would offer valuable insights into the recovery process and the efficacy of different intervention strategies in restoring physical fitness levels. These studies should also explore the long-term psychological impacts and behavioral adaptations resulting from extended periods of physical inactivity. Lastly, while our findings underscore the need for targeted interventions to mitigate the negative effects of pandemic-related disruptions on adolescent physical fitness, specific recommendations for intervention programs or policy measures were not sufficiently detailed in our initial analysis. Future studies should explore the effectiveness of various physical activity programs and policies in promoting physical fitness during times of social restrictions. Such research could inform public health strategies and educational policies aimed at maintaining and enhancing physical fitness among adolescents in the face of future public health crises.

## Conclusion

In conclusion, our study provides a detailed examination of the significant impacts of the COVID-19 pandemic on the physical fitness of high school students, distinguishing between those engaged in regular athletic activities and their sedentary counterparts. The findings underscore a notable decline in physical fitness levels, particularly among athletes, highlighting the profound effects of pandemic-induced disruptions on structured physical activities and training routines. Moreover, the diminished discriminative power of the ALPHA test battery post-pandemic signals a need for continuous monitoring and assessment of physical fitness measures to better understand and address the evolving challenges faced by youth in maintaining physical health. Future research should expand upon our findings by incorporating diverse populations, employing objective measures of physical activity, and exploring the interrelations between physical, psychological, and behavioral health aspects. This holistic approach will enable a more comprehensive understanding of the pandemic’s multifaceted impact on youth and inform the development of effective, evidence-based interventions. Ultimately, our study calls for an integrated effort among educators, policymakers, and health professionals to prioritize and promote physical activity as a crucial component of youth development and public health, particularly in the face of challenges posed by the COVID-19 pandemic and potential future public health emergencies.

## Data Availability

The datasets generated and analyzed during the current study consist of numerical data related to physical fitness measurements. These data are available upon reasonable request. Requests for access to these data should be directed to Dr. S.U. (suleyman.ulupinar@erzurum.edu.tr). Data are provided under specific conditions which will be detailed upon request. The numerical data are primarily in Excel format. Supplementary documentation, including a data dictionary explaining the measurements and a methodology guide detailing the data collection and analysis procedures, is also available to facilitate understanding and use of the dataset.

## Abbreviations

BMI: Body Mass Index
COVID-19: Coronavirus Disease 2019
ALPHA: Assessing Levels of Physical Activity
VO_2max_: Maximum Oxygen Uptake
SD: Standard Deviation
ANOVA: Analysis of Variance
SPSS: Statistical Package for the Social Sciences
*p*-value: Probability Value
t-test: Student’s t-Test
F-value: F Statistic
r: Pearson Correlation Coefficient
df: Degrees of Freedom
